# Ganglion Cell Adaptability: Does the Coupling of Horizontal Cells Play a Role?

**DOI:** 10.1371/journal.pone.0001714

**Published:** 2008-03-05

**Authors:** Karin Dedek, Chethan Pandarinath, Nazia M. Alam, Kerstin Wellershaus, Timm Schubert, Klaus Willecke, Glen T. Prusky, Reto Weiler, Sheila Nirenberg

**Affiliations:** 1 Department of Neurobiology, University of Oldenburg, Oldenburg, Germany; 2 Department of Physiology and Biophysics, Weill Medical College of Cornell University, New York, New York, United States of America; 3 Canadian Centre for Behavioural Neuroscience, The University of Lethbridge, Lethbridge, Alberta, Canada; 4 Institute of Genetics, University of Bonn, Bonn, Germany; Oregon Health & Science University, United States of America

## Abstract

**Background:**

The visual system can adjust itself to different visual environments. One of the most well known examples of this is the shift in spatial tuning that occurs in retinal ganglion cells with the change from night to day vision. This shift is thought to be produced by a change in the ganglion cell receptive field surround, mediated by a decrease in the coupling of horizontal cells.

**Methodology/Principal Findings:**

To test this hypothesis, we used a transgenic mouse line, a connexin57-deficient line, in which horizontal cell coupling was abolished. Measurements, both at the ganglion cell level and the level of behavioral performance, showed no differences between wild-type retinas and retinas with decoupled horizontal cells from connexin57-deficient mice.

**Conclusion/Significance:**

This analysis showed that the coupling and uncoupling of horizontal cells does not play a dominant role in spatial tuning and its adjustability to night and day light conditions. Instead, our data suggest that another mechanism, likely arising in the inner retina, must be responsible.

## Introduction

Spatial tuning is a fundamental feature of retinal ganglion cells. It allows the detection of spatial patterns on multiple scales [1]–[3]. Some cells, for example, are tuned to low spatial frequencies and allow the detection of large spatial patterns. Others are tuned to high spatial frequencies and permit the resolution of fine details (reviewed in [2]).

A ganglion cell's sensitivity to spatial patterns is a function of its receptive field organization. Most ganglion cell receptive fields consist of two components, a center and a surround that respond oppositely to light [4], [5]. What tunes a ganglion cell to a particular spatial scale are the sizes of these two components and their relative strengths [2], [3], [6].

Though the organization of the ganglion cell receptive field has been known for decades, the mechanisms that generate it are not completely understood. The center response is thought to result from vertical signaling from photoreceptors to bipolar cells to ganglion cells. The origin of the surround response, however, is controversial. Early reports suggested that it was generated by horizontal cells [7], [8] which appear to act through two pathways: feedback inhibition to photoreceptors [9], [10] and feedforward inhibition to bipolar cells [11], [12]. More recent studies, however, indicate a contribution from amacrine cells [6], [13]–[16] which also employ two distinct pathways: direct input to ganglion cells [13], [14], [16] and feedback signaling onto bipolar cell terminals [16]. The relative contributions of these four different surround-generating mechanisms are unclear and remain a subject of much discussion [6], [13], [15]–[17].

A key aspect of the discussion concerns one of the most intriguing features of spatial tuning–its adjustability. It is well known that the spatial tuning can adjust itself in the face of different visual environments [18], [19]. The most well known example is the shift in tuning that occurs when the retina moves from the dark-adapted to the light-adapted state (from night to day vision) [18], [20]–[22]. It has long been proposed that this shift is caused by a change in the ganglion cell receptive field surround, mediated by a change in the coupling of horizontal cells [23]. This conjecture arose because this coupling is known to vary with ambient light intensity [23]–[26].

**Figure 1:**
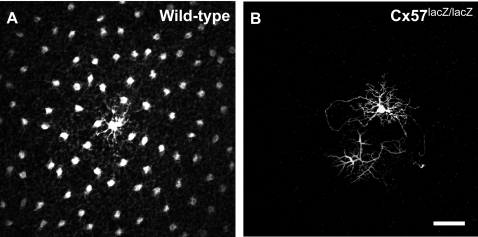
Dye coupling was abolished in Cx57-deficient mice. (A) Neurobiotin-injected horizontal cells from wild-type mice showed extensive coupling. Note that coupling extended beyond the borders of the image. In total, 182 horizontal cells were coupled to this cell. (B) Horizontal cell from a Cx57-deficient mouse, injected under the same conditions. Coupling was abolished in these mice. Similar results have been shown before [27], [28]. Scale bar, 50 µm.

To test this hypothesis, we used a transgenic mouse line, a Connexin57-deficient line, in which horizontal cell coupling is more than 99% abolished, as measured by dye-transfer (Fig. 1; [27], [28]). *Connexin57* (*Cx57*), a gene that encodes a gap junction protein, is exclusively expressed in retinal horizontal cells, so no other cell classes are affected [27], making this mouse line a powerful model to very selectively address this question.

The results showed that the coupling and uncoupling of horizontal cells does not play a critical role in spatial tuning, that is, it does not substantially contribute to the mechanism that controls the changes in spatial tuning that occur with the switch from night to day vision. We tested this both at the level of ganglion cell performance, using spatial tuning curves and center-surround measurements, and at the level of behavioral performance, using visual psychometric measurements. This analysis provides strong evidence that another mechanism has to be responsible.

## Results

Our aim was to test the hypothesis that changes in horizontal cell coupling play a role in ganglion cell spatial tuning, in particular, in the shift in tuning that occurs when animals move from daytime to nighttime viewing conditions. For that purpose we used a Cx57-deficient mouse line, generated in a C57BL/6 background, and compared it to wild-type C57BL/6 mice, in which horizontal cell coupling was unperturbed (Fig. 1). In both mouse lines, spatial tuning and its adjustability were evaluated at the ganglion cell level and the level of behavioral performance.

### Ganglion cells from wild-type and Cx57-deficient mice showed the same shift in spatial tuning

To evaluate ganglion cell spatial tuning, standard methods were used [1], [6], [29], [30]. Briefly, drifting sine wave gratings of different spatial frequencies were projected onto the retina, and ganglion cell responses were recorded extracellularly. A spatial tuning curve for each cell was then generated by Fourier analyzing the responses and plotting the amplitude of the fundamental as a function of spatial frequency. To assess the adjustability of spatial tuning, the gratings were presented at two different light intensities, one scotopic and one photopic (see *Methods* for the intensities).

**Figure 2:**
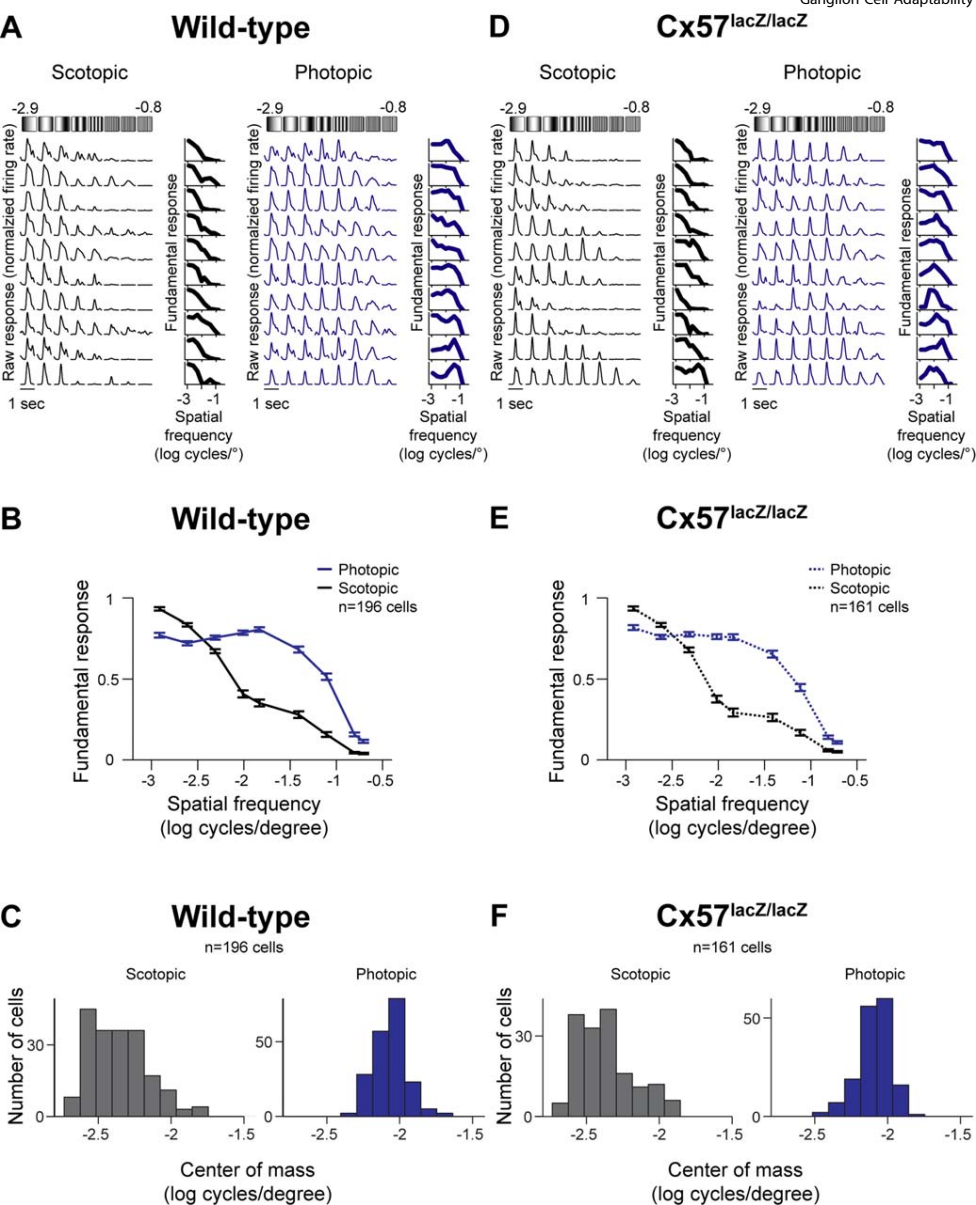
Ganglion cells from wild-type and Cx57-deficient mice showed the same shift in spatial tuning. (A, D) Representative ganglion cell responses from wild-type (A) and Cx57-deficient (D) mice to drifting sine wave gratings presented at two different light intensities: scotopic, grey, and photopic, blue. See *Methods*, for light intensities. Responses were normalized to the maximum firing rate. Each cell's tuning curve is presented at the right. (B, E) Average tuning curves (mean±SEM) for all cells from wild-type (B) and Cx57-deficient (E) retinas, measured at the scotopic (grey) and the photopic (blue) light intensities. (C, F) Distribution of the center of mass values for all cells from wild-type (C) and Cx57-deficient (F) retinas measured at the scotopic (grey) and the photopic (blue) light intensities. No significant difference was observed between the two genotypes for the scotopic condition (*p*>0.35, *n* = 196 cells for wild-type, *n* = 161 for Cx57-deficient, *KS* test) or the photopic condition (*p*>0.18, *n* = 196 for wild-type, *n* = 161 for Cx57-deficient, *KS* test).

Consistent with studies performed in other species [1], [31], [32], wild-type mouse retinal ganglion cells showed a shift in spatial tuning when the light level was changed from scotopic to photopic. Specifically, the weight of the ganglion cells' tuning curves shifted from low spatial frequencies toward high. Figure 2A shows representative examples; Figure 2B shows the mean for all 196 cells in the dataset.

To quantify the shift, a center of mass analysis was performed, following Sinclair et al. [6]. At each light level, the center of mass of each tuning curve was calculated, and the distribution of center of mass values was plotted (Fig. 2C). This analysis showed a very statistically significant difference between the two distributions [*p<*10^−4^, *Kolmogorov-Smirnov (KS)* test]; the mean center of mass value for the photopic condition was nearly twice the spatial frequency of the mean center of mass value for the scotopic condition.

To test whether changes in horizontal cell coupling play a role in mediating this shift, we compared the tuning curves produced by Cx57-deficient retinas, that is, retinas in which the horizontal cell coupling was reduced by >99%, with those from the normal, wild-type retinas. If changes in the coupling play a role, then there should be no shift in the Cx57-deficient retinas. The results showed that this was not the case. Just as in the wild-type retinas, the tuning curves from the Cx57-deficient mice were weighted towards low spatial frequencies in the scotopic light condition and toward high spatial frequencies in the photopic light condition. Representative examples are shown in Figure 2D; for each cell, the left column shows the tuning curve at the scotopic light level and the right column shows the tuning curve for the same cell at the photopic light level. Figure 2E shows the mean tuning curves for all 161 cells in the dataset. When the shift was quantified using the center of mass analysis (Fig. 2F), the results showed no significant difference between the tuning curves from the Cx57-deficient and normal, wild-type retinas (*p*>0.35, *KS* test, comparing the distribution of center of mass values from the Cx57-deficient retinas taken at the scotopic light level with the same from the wild-type retinas, and *p*>0.18, comparing the distribution of center of mass values from the Cx57-deficient retinas taken at the photopic light level with the same from the wild-type retinas).

In summary, these results show that retinas with coupled and uncoupled horizontal cell networks undergo the same shift in spatial tuning when light levels change from scotopic to photopic conditions.

### Ganglion cells from wild-type and Cx57-deficient mice had the same surround size

**Figure 3:**
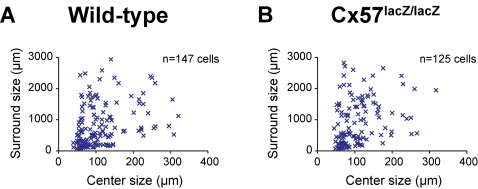
The distributions of surround sizes were the same for both genotypes. Ganglion cells from (A) wild-type and (B) Cx57-deficient mice (*p*>0.64, *n* = 147 for wild-type, *n* = 125 for Cx57-deficient, *t-*test) had the same surround sizes [measured only at the photopic level, as there is little or no surround at the scotopic level for both genotypes (see text and *Methods*)].

The center of mass analysis shows that the spatial tuning curves undergo a shift with the change in light intensity, but it does not show where in the curves the shift occurs. Because the shift could be caused by any change in the center/surround organization of the ganglion cell receptive field [2], [6], [33], we tested specifically whether it was due to a change in the surround, as expected from previous studies in other species [18], [32]. To test this, we fit the tuning curves to a standard receptive field model, a difference of Gaussians model [6], [34], and measured surround size. Consistent with the studies in other species [18], [32], the receptive fields showed no surrounds at the scotopic light level (>80% of cells were better fit by a single Gaussian, see *Methods*), but gained surrounds in photopic light with a mean surround size of 972±78 µm (*n* = 147) (Fig. 3A). Thus in the wild type, the observed shift in the spatial tuning curves upon light intensity increase was accompanied by a gain of surround (see refs. 33 and 35 for detailed quantitative analysis of how center and surround parameters affect spatial tuning curves; for further discussion, see [6]).

To test whether changes in the coupling of horizontal cells play a critical role in mediating this gain, we compared ganglion cell surround sizes from Cx57-deficient retinas with those from wild-type retinas. If changes in the coupling play a strong role, then surround sizes should be different in the two genotypes. Our results indicate that this was not the case. As in the wild-type retinas, ganglion cells from the Cx57-deficient retinas showed no surrounds at the scotopic light level and gained surrounds in photopic light (Fig. 3B), and there was no significant difference in the surround size (mean surround size in the Cx57-deficient retinas was 1022±76 µm, *n* = 125, compared to 972±78 µm, *n* = 147 in the wild-type retina, *p*>0.64, *t-*test).

In sum, ganglion cells from wild-type mice showed a shift in the weight of the spatial tuning curves when the ambient light was increased from scotopic to photopic levels. This shift was associated with a gain in surround size. Ganglion cells from Cx57-deficient mice showed essentially the same behavior (no statistically significant difference), providing further evidence that the coupling and uncoupling of horizontal cells is not the critical mechanism that underlies the change in spatial tuning that occurs with different light intensities.

### Spatial tuning was similar in behaving wild-type and Cx57-deficient mice

To assess the role of horizontal cell coupling in spatial tuning on a larger scale, we compared the behavioral performance in spatial pattern detection for wild-type and Cx57-deficient mice using psychometric measurements. For this purpose we used a virtual optokinetic system that allowed a rapid analysis of visual thresholds in freely moving mice [36], [37]. Animals from both genotypes were presented with drifting sine wave gratings of decreasing contrast to determine contrast sensitivity at a given grating spatial frequency. Since the optokinetic task is not suitable to test visual performance in the low spatial frequency range, only gratings that had a spatial frequency of at least 0.05 cycles/degree were presented.

**Figure 4:**
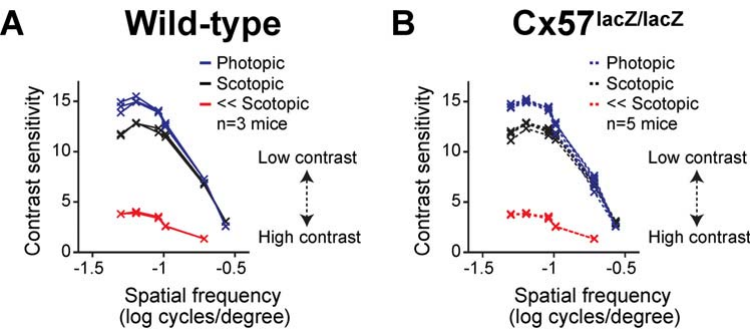
Visual performance, measured as contrast sensitivity, was the same for both genotypes. For (A) wild-type and (B) Cx57-deficient mice, measurements were taken at three different light intensities: *blue*, photopic, *black*, scotopic, *red*, low scotopic; see *Methods* for all light intensities (*p>*0.25, low scotopic: *p>*0.5, scotopic: *p>*0.1, photopic, *t-*test, Bonferroni corrected). Contrast sensitivity in all mice showed an increase in amplitude and a broadening of spatial frequency profile with increases in light intensity. Note that these are threshold measurements, rather than averages, as described in [36], [37]. With these measurements, animals are pushed to their best performance, which reduces animal-to-animal variability that arises from unrelated causes (e.g., differences in learning or inattention).

As shown before in the mouse and other species [31], [38], [39], in wild-type mice, contrast sensitivity was lower under scotopic than under photopic conditions. At the higher light intensity, the mice needed less contrast to track the grating (Fig. 4A). If horizontal cell coupling does not control spatial tuning at the behavioral level, then performance on this task for the Cx57-deficient and wild-type mice should not differ. This was indeed the case (Fig. 4B). Performance was not significantly different between the wild-type and Cx57-deficient animals under both scotopic (*p*>0.5, *t*-test, Bonferroni corrected) and photopic (*p*>0.1, *t*-test, Bonferroni corrected) light conditions. Note that the behavioral measurements shown in Figure 4 are threshold measurements, rather than averages, following Prusky et al. [36] and Douglas et al. [37]. With these measurements, animals are pushed to their best performance, which reduces animal-to-animal variability that arises from unrelated causes (e.g., differences in learning or inattention).

To push the system further, we repeated the psychometric measurements at a much lower light level (4.5 orders of magnitude lower; see *Methods* for all light intensities used). At this intensity, contrast sensitivity was much lower and had a smaller profile than at the intensities used before (Fig. 4A, red line). However, behavioral performance from mice lacking horizontal cell coupling was the same as in wild-type mice (Fig. 4B, *p*>0.25, *t*-test, Bonferroni corrected). Thus, at all the light intensities tested (again, see *Methods* for all intensities) contrast sensitivity between wild-type and Cx57-deficient mice was not significantly different.

### Verification with dopamine

**Figure 5:**
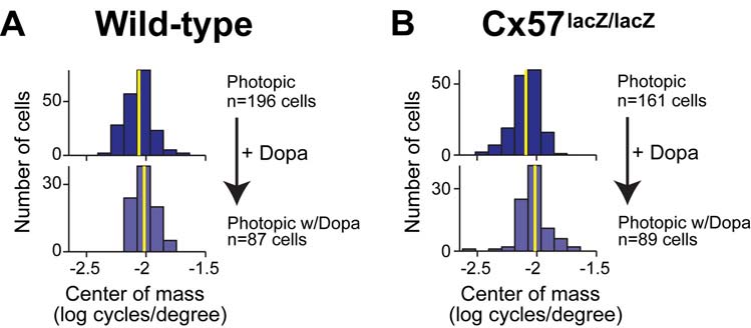
The shift produced by dopamine was the same for both genotypes. For (A) wild-type and (B) Cx57-deficient retinas, the center of mass values showed a shift in tuning towards higher spatial frequencies with the addition of dopamine (*p<*0.0011 for wild type, *n* = 196 control, *n* = 87 dopamine; *p<*0.0016 for Cx57*-*deficient, *n* = 161 control, *n* = 89 dopamine, *KS* test*).* Yellow lines indicate the mean of the distributions to clarify the shift. There were no significant differences between the two genotypes (*p>*0.16 without dopamine, *n* = 196 wild-type, *n* = 161 Cx57-deficient; *p>*0.77 with dopamine, *n* = 87 wild-type, *n* = 89 Cx57-deficient, *KS* test).

To further assess the result that the coupling of horizontal cells does not play a strong role in ganglion cell spatial tuning, we perturbed horizontal cell networks with the neuromodulator dopamine. Dopamine has been shown to affect ganglion cell receptive fields and therefore the spatial tuning of ganglion cells [40], [41] although there is some disagreement about this [42]. The mechanism by which it acts is not known since dopamine operates at multiple sites within the retina, but the most widely hypothesized mechanism involves actions on the coupling and uncoupling of horizontal cells [43]–[45]. If dopamine's effects on ganglion cell spatial tuning are mediated through changes in horizontal cell coupling, then its actions should be different in Cx57-deficient versus wild-type mice. We tested this under photopic conditions (note that the coupling in wild-type retinas even under photopic conditions is still higher by a factor of at least 100 compared to the 99% abolished coupling in the Cx57-knockout [27], [28]), and our results showed that this was not the case. Consistent with expectation [28], [46], [47], dopamine (100 µM) applied to wild-type retinas produced a shift in the weight of the ganglion cell tuning curves toward higher spatial frequencies (Fig. 5A, *p<*0.0011, *KS* test; data are also presented as average tuning curves in *Supp. Info.* Fig. S2a). When the same concentration of dopamine was applied to Cx57-deficient mice, the same shift was observed (Fig. 5B), *p<*0.0016, *KS* test). When the two shifts were compared, there was no statistically significant difference (*p>*0.77, *KS* test). Since the shift in ganglion cell spatial tuning occurred in the Cx57-deficient mice, it has to be mediated by a process other than a change in horizontal cell coupling.

### Blocking the feedback from horizontal cells to photoreceptors altered spatial tuning

**Figure 6:**
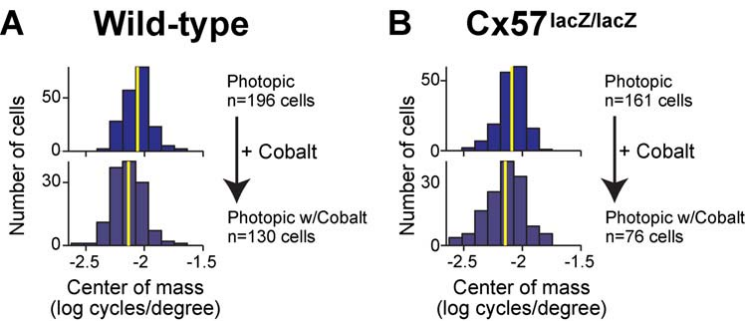
The shift produced by cobalt was the same for both genotypes. For (A) wild-type and (B) Cx57-deficient retinas, the center of mass values showed a shift in tuning towards lower spatial frequencies with the addition of cobalt (*p<*2×10^−5 ^for wild type, *n* = 196 control, *n* = 130 cobalt; *p<*0.0019 for Cx57*-*deficient, *n* = 161 control, *n* = 76 cobalt, *KS* test*).* Yellow lines indicate the mean of the distributions to clarify the shift. There were no significant differences between genotypes (*p>*0.16 without cobalt, *n* = 196 wild-type, *n* = 161 Cx57-deficient; *p>*0.66 with cobalt, *n* = 130 wild-type, *n* = 76 Cx57-deficient, *KS* test).

Since our experiments did not show a role for the coupling of horizontal cells in ganglion cell spatial tuning and its adjustability, the question arises whether horizontal cells contribute to ganglion cell spatial tuning at all in the mouse. Horizontal cells provide negative feedback to cone photoreceptors [48] which has been shown in other species to play a role in the organization of ganglion cell receptive fields [10], [11], [42], [48], [49]. Feedback can be blocked with cobalt at submillimolar levels (100 µM). At this concentration, feedforward signaling from cones to horizontal cells is intact [50], but negative feedback from horizontal cells to cones is attenuated [42], [51]. As with dopamine, we tested the effect of cobalt under photopic conditions. If horizontal cell feedback is involved in spatial tuning, then tuning should be shifted towards lower spatial frequencies in the presence of cobalt compared to control conditions. Indeed, this was the case (Fig. 6). As expected [10], application of 100 µM cobalt to the wild-type retina led to a shift in spatial tuning towards lower spatial frequencies (Fig. 6A, *p<*2×10^−5^, *KS* test (data are also presented as average tuning curves in *Supp. Info*. Fig. S2b). In line with previous reports from other species [42], [48], this indicates that negative feedback from horizontal cells to photoreceptors contributes to the spatial tuning of ganglion cells. In Cx57-deficient mice, application of cobalt led to the same shift in spatial tuning (Fig. 6B, *p<*0.0019, *KS* test) in a way that was not significantly different from its effect in wild-type mice (p>0.66, *KS* test). This indicates that feedback was intact in Cx57-deficient mice.

Note that, even when horizontal cell coupling was abolished and horizontal cell feedback was inhibited, spatial tuning under photopic conditions was shifted towards higher spatial frequencies than under scotopic conditions (compare Fig. 6B with Fig. 2F, *p<*10^−4^, *KS* test). This suggests that either cobalt does not completely block horizontal cell feedback in the mouse or that processes in the inner retina must be contributing to spatial tuning.

## Discussion

Numerous studies have shown, at the behavioral level, that the visual system can adjust itself to different visual environments [18], [19], [20]–[22], [52]–[54]. One of the most well known examples of this is the shift in spatial frequency sensitivity that occurs with the change from night (scotopic) to day (photopic) vision [18], [20]–[22], [31], [39], [55]. This shift serves presumably as an information-optimizing strategy: at night, i.e., under photon-limited conditions, where the signal-to-noise ratio is low, the visual system is better served by integrating over a large area, so it shifts its tuning toward low spatial frequencies. During the day, when photons are not limiting, the system is better served by integrating over smaller areas, so it can resolve image details; in this case, the shift is toward high spatial frequencies (reviewed in [56], [57]).

How the visual system performs this shift is not clear. A large body of evidence, though, points to the retina as the starting point since the shift is detectable at the level of the ganglion cells [18], [21], [32]. What remains to be determined is the mechanism that confers this on the cells. The most likely candidate is a change in the surround component of the ganglion cell's receptive field, as it is the surround that shapes the amplitude of the ganglion cell's response at low spatial frequencies. Changes in surround size cause the cell to shift its response toward or away from low spatial frequencies (see refs. 33, 35, and 6 for detailed quantitative analysis of how center and surround parameters affect the shape of the ganglion spatial tuning curve).

A long-standing proposal for how surround size might change with different light levels is that it might do so through a change in the gap junctional coupling of horizontal cells. The rationale for this hypothesis is that the extent of horizontal cell coupling is dependent on ambient light intensity [24]–[26]. Thus, a change in horizontal cell coupling can serve as a natural knob for adjusting surround size and, therefore, the spatial tuning of the ganglion cells.

Here we tested this proposal. We used the mouse as a model system. We first measured ganglion cell spatial tuning at scotopic and photopic light levels in wild-type animals. As expected, the tuning shifted from low to high spatial frequencies as light intensities were increased from a lower to a higher level. We then measured the spatial tuning in Cx57-deficient mice in which horizontal cell coupling was reduced by >99%. If horizontal cell coupling plays a critical role in the adjustability of ganglion cell spatial tuning, then the shift from low to high spatial frequencies should be abolished. Our results indicated that it was not (Fig. 2). The shift from low to high spatial frequencies was essentially identical to that observed in wild-type mice. Direct measurements of ganglion cell surround size then confirmed this: If horizontal cell coupling plays a major role in the adjustability of ganglion cell surround size, then the shift from “no surround” to “small surround” should be abolished. It wasn't. The shift was essentially identical to that observed in the wild type (Fig. 3). Finally, behavior measurements provided further confirmation. No difference in spatial tuning sensitivity was observed between the Cx57-deficient and wild-type animals (Fig. 4).

These results thus provide strong evidence that changes in the coupling of horizontal cells is not a dominant mechanism for controlling the spatial tuning of ganglion cells. Most significantly, it does not appear to be a critical player in the adjustability of the tuning that occurs with changes from night to day vision. Other processes must dominate. Our measurements with dopamine confirmed this: dopamine's effects on the spatial tuning of ganglion cells could not have been mediated by a change in horizontal cell coupling since dopamine led to the same shift in spatial tuning in Cx57-deficient mice as in wild type, at least under photopic conditions (Fig. 5). This raises the idea that dopamine's dominant effects with respect to spatial tuning are on other retinal pathways e.g., affecting other electrically coupled networks in the retina [58], most likely amacrine cell networks [59].

In sum, with the aid of a *Connexin57* knock out, we were able to test the long-standing hypothesis that the coupling and uncoupling of horizontal cells serves as a critical knob for adjusting spatial tuning to different light conditions, i.e., to night versus day conditions. The results show that this hypothesis, at least as it currently stands, must be rejected. The evidence for rejection is extremely strong because the same result presented itself at multiple levels–that is, when changes in horizontal cell coupling were prevented, as was the case in the knock out, the shift in spatial tuning that occurs when the retina moves from night to day proceeded normally–as measured at the level of both ganglion cell performance and whole animal behavioral performance. Thus, changes in horizontal cell coupling cannot be the critical mechanism that underlies this shift.

At first glance, it might seem surprising that preventing the changes in horizontal cell coupling–an act that affects lateral signaling in the retina–had no significant effect on ganglion cell spatial tuning, but this result can be reconciled with the many recent reports that this tuning is shaped by more than one set of circuits–that is, it is shaped by circuits in both the outer and inner retina [6], [13], [15]–[17], [48]. What the results of our experiments suggest is that inner retinal circuits dominate–at least for the problem of adjusting spatial tuning to different light conditions. Whatever occurs when the horizontal cells change from the uncoupled to the coupled state is effectively swamped by stronger circuit actions that occur in the inner retina.

This raises the intriguing question of what the changes in horizontal cell coupling are for. One possibility is that they serve to facilitate signal detection in the time domain, rather than the space domain. A change in horizontal cell coupling, because it is a change in the state of a potential shunt [60], would be expected to affect both spatial and temporal signal detection. If its effects on spatial signal detection are redundant to those produced by the inner retina, then losing the coupling would have minimal effect on spatial processing. If its effects on *temporal* signal detection are not redundant, then losing it should affect temporal processing. This work thus creates a new hypothesis for the function of the horizontal cell coupling–that it serves to improve signal-to-noise ratios in the time domain, and, therefore, may be a key player in temporal processing.

Finally, for the sake of completeness, we conclude by stating that we cannot completely rule out the possibility that there is another connexin that links horizontal cells. However, if one exists, the likelihood that it contributes substantially to horizontal cell coupling is very small. The reason we state this is that the effects of knocking out Cx57 on horizontal cell coupling are maximal or near maximal, as measured by changes in both dye coupling and horizontal cell length constant. Dye coupling, using neurobiotin, is >99% abolished [27], [28], and horizontal cell length constants are significantly reduced [28], with a reduction greater than that produced by dopamine application, which also reduces horizontal cell coupling (the hierarchy of length constant reduction is shown in *Supp. Info.* Fig. S3). With respect to receptive field evaluations: horizontal cell length constants in the knockout are on average 50 µm, with the mean dendritic tree diameter for individual horizontal cells at 100 µm [28]. Taken together, these data provide strong evidence that Cx57 is the primary, or exclusive, mediator of horizontal cell coupling, and that eliminating its ability to function provides a strong test for the role of horizontal cell coupling in retinal processing.

## Materials and Methods

### Animals

For generation of the *Cx57-lacZ* mouse line, part of the coding region of the *Cx57* gene was deleted and replaced with the *lacZ* reporter gene [27]. Cx57-deficient mice (Cx57*^lacZ^* ^/*lacZ*^) and wild-type controls aged 2 to 4 months were used for all experiments. After each recording, the genotype of the retina was confirmed with staining for β-galactosidase activity and PCR as described [27]. All experiments were conducted in accordance with the institutional guidelines for animal welfare.

### Extracellular recordings of ganglion cell responses

The isolated mouse retina was placed on a flat array of 64 microelectrodes as described [6] and bathed in oxygenated Ringer's solution at room temperature. Recordings were made from central retina as described previously [6], [61]. Briefly, spike trains were recorded using a Plexon Instruments Multichannel Neuronal Acquisition Processor (Dallas, TX). A custom made time-voltage window discriminator that captured distinct waveforms served to sort spikes on-line into individual units.

### Light stimulation

An overhead projector (EIKI OHP-4100, Rancho Santa Margarita, CA) in combination with a liquid crystal display panel (Panasonic PT-L104, Secaucus, NJ) was used to deliver visual stimuli. Neutral density filters attenuated the stimulus intensity to the desired scotopic and photopic light levels. The scotopic intensity was 0.0066 µW/cm^2^; the photopic was 0.21 µW/cm^2^. Following ref. 62, and using the spectrum of our monitor, also available in ref. 61, these radiometric units can be converted to photoreceptor equivalent photons/µm^2^/s: The scotopic intensity converts to 52.5 rod-equivalent photons/µm^2^/s and 60 M-cone-equivalent-photons/µm^2^/s, the photopic, to 1670 rod-equivalent-photons/µm^2^/s and 1900 M-cone-equivalent-photons/µm^2^/s. This gives a rate of 32.5 R*/rod/s and 21 R*/M-cone/s for scotopic, and 1120 R*/rod/s, and 650 R*/M-cone/s for photopic, assuming an effective collecting area (i.e., collecting area/funneling factor) from [62], [63] of 0.67 µm^2^ for rods and 0.34 µm^2^ for cones. Note that the emphasis here is on rods and M-cones, as UV pigments are not significantly stimulated with the displays presented in this paper.

As mentioned in the *Introduction* and *Results*, these light levels were chosen to bring out the shift in spatial tuning that occurs as the retina moves from night to day vision, as shown in Fig. 2, and to span the range where changes in horizontal cell coupling are maximal or near maximal, as observed in both mouse [64] and rabbit [26, Figs. 5 and 9]. The scotopic and photopic light levels are also consistent with the levels reported for the mouse rod and cone regimes, as assessed using rod saturation measurements, by Dodd [65].

All stimuli used white light (for spectrum, see [61]) and consisted of random flicker, flashes and gratings. To measure receptive field properties of ganglion cells, we used drifting sine wave gratings with 8 different spatial frequencies ranging from 10^−2.9^ to 10^−0.8^ = 0.0012 to 0.155 cycles/degree in three directions. Each spatial frequency and direction was presented for 30 cycles, with a temporal frequency of 1 Hz. The 24 combinations of spatial frequency and direction were randomly interleaved. Measurements always started at the scotopic light intensity. After increasing the light intensity, a series of flashes was run which was followed by a random flicker stimulus to adapt the retina for 20 min before the grating stimulus was started. For the experiments involving drugs (dopamine and cobalt), the drugs were applied during this adaptation time.

### Pharmacology

All chemicals were purchased from Sigma (St. Louis, MO). Cobalt and dopamine were dissolved in oxygenated Ringer's solution and were delivered to the retina by continuous perfusion.

### Data analysis

Spatial frequency analysis was done using standard methods [6]. Briefly, the spatial tuning of each ganglion cell was evaluated using its responses to drifting sine wave gratings of varying spatial frequency and direction (8 spatial frequencies, 3 directions, see above). For each grating, the first harmonic of the response was calculated. The first harmonic, *R*(**k**), with **k** = (*k_x_*,*k_y_*) as the two-dimensional spatial frequency, was computed as follows:
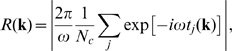
where *ω = 2π* radians/s is the temporal frequency of the drifting sine wave grating; *N_c_* is the number of cycles (30 in our experiments); and *t_j_* (**k**) is the time of the *j*th spike produced by a grating with the spatial frequency **k**. Tuning curves, which give *R*(***k***) as a function of ***k***, were then plotted.

To determine the mean of each cell's spatial tuning curve, the center of mass (CM) of the curve was calculated as:
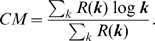



To determine the center-surround receptive field parameters for each cell, the cells' tuning curves were fit with the standard difference-of-Gaussians model. The model linearly combines the profiles of a tall and narrow Gaussian representing the center and a short and shallow Gaussian of opposite sign representing the surround (see [6], [34]; we followed [6] directly). The model is based on seven parameters; to determine the values of the parameters that give the best fit to the curve, the mean squared error between *R*(**k**) and the response predicted by the model 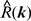 was minimized, using a brute force exploration of initial conditions to find the global minimum. 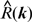 was calculated as:
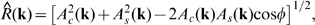
where
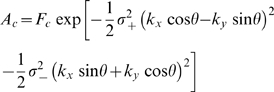
is the strength of the center response, *σ_+_* and *σ_−_* the major and minor radii of the center (assumed to be asymmetric, based on [6]), *θ* its orientation, and
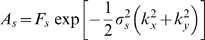
is the strength of the surround response, where *σ_s_* (assumed to be symmetric, also based on [6]) is the size of the surround, and *φ* the phase angle associated with the different delays between the center and surround response. The mean squared error between *R*(**k**) and 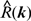, denoted *χ* ^2^, is given by:
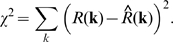



Goodness of fit was then measured by *r^2^*, the fraction of the variance explained by the model, where *r^2^* = 1−*χ* ^2^/Var[*R*(k)]. Following [6], only cells whose *r^2^* values were >0.6 were included in the dataset. (For visualization of the quality of an *r^2^* value of 0.6, a hierarchy of fits from *r^2^*>0.9 to *r^2^*<0.6 is shown in *Supp. Info.*, Fig. S1.) Also following [6], for each parameter, only parameter values that were within 3 standard deviations of the mean for that parameter were included.

#### Receptive fields with no surrounds

At scotopic light levels, ganglion cell receptive fields showed no surrounds, that is, the best fit, as measured by *r^2^* was a single Gaussian; no increase in *r^2^* of more than 0.05 was achieved by including a second Gaussian. >80% of cells at scotopic light levels fell into this category. For a clear demonstration that the single Gaussian was the better fit, see Figure 2: As shown in panels B and E, as well as in panels A and C, most (>80%) of the tuning curves at scotopic light levels (black curves) are monotonically decreasing; this is consistent with a fit to a single Gaussian.

### Behavioral tests using a virtual optokinetic system, light intensities

Responses were measured using the Prusky/Douglas virtual optokinetic system [36], [37]. Briefly, the animal, which was freely moving, was placed in a virtual reality chamber, a virtual cylinder, that projects a vertical sine wave grating. A video camera, situated above the animal, provided live video feedback of the testing arena. The walls of the cylinder were kept a constant distance from the animal's head, “clamping” the spatial frequency of the grating. On each trial, the cylinder was centered on the mouse's head. A drifting grating of a pre-selected spatial frequency at 100% contrast appeared, and the mouse was assessed for tracking behavior for a few seconds. Grating contrast was systematically reduced until no tracking response was observed. The data were then evaluated by fitting the animal's response to steps of decreasing contrast to a logistic function (a psychometric function) using the psignifit, version 2.5.6 for Matlab which implements the maximum-likelihood method described by Wichmann and Hill [66]. The animal's contrast threshold for each spatial frequency was taken as the 50% point of the fitted curve. Contrast was calculated from the gratings luminances on the screen: (Lmax–Lmin)/(Lmax+Lmin). Contrast sensitivity is the reciprocal of the threshold. Significance testing was performed for each light level using *t*-tests with Bonferroni correction for multiple comparisons.

Light intensities for the behavior experiments were measured in cd/m^2^ using a luminance meter (Minolta, model LS-100). Three were used: 17.9 cd/m^2^, 0.6 cd/m^2^ and 1.8×10^−5^ cd/m^2^. Following ref. 63, which provides a conversion from cd/m^2 ^to photoreceptor-equivalent photons/µm^2^ for mouse, and adjusting for pupil size as in [67] (Figs. 2 and 3), these intensities cover the same range as those used in the recording chamber: 1640 rod-equivalent-photons/µm^2^/s (0.5 mm^2^ pupil area), 350 rod-equivalent-photons/µm^2^/s (fully dilated pupil) to <0.1 rod-equivalent-photons/µm^2^/s. Note that mice have substantial vision at very low light levels, as measured by electroretinogram and optomotor responses (see ref. 68, Figs. 2 and 3 and ref. 69, Fig. 7).

## Supporting Information

Figure S1 As indicated in the main text (*Methods*), for quality control, and for consistency with previous work [6], only fits with r^2^ values >0.6 were used. To provide intuition for the quality of an r^2^ value of >0.6, a series of fits from r^2^>0.9 to r^2^<0.6 is shown. A natural breakdown begins below 0.6. Data are plotted on semi-log plots; *red dots* indicate cells' responses, *blue curves* indicate fits.
(0.31 MB TIF)

Figure S2 Shifts in spatial tuning following dopamine and cobalt application, presented as average tuning curves. In the main text, the shifts were presented as center-of-mass distributions; that is, we took each cell's tuning curve, measured its center of mass and presented the distribution of center of mass values for all cells in the data set (see Figs. 5 and 6). For the interested reader, we show here the shifts as average tuning curves (mean±SEM); *arrows* indicate direction of shift. Consistent with the center of mass analysis, where all significance tests are presented, dopamine causes a shift to the right for both genotypes, and cobalt causes a shift to the left for both genotypes. *Blue* indicates no drug; *red* indicates drug.
(0.38 MB TIF)

Figure S3 Horizontal cell length constants in the Cx57-deficient mice are significantly reduced. As indicated in the main text, the evidence that knocking out Cx57 blocks horizontal cell coupling is that dye spread (neurobiotin) is >99% abolished, and horizontal cell length constants are significantly reduced, with the reduction greater than that produced by dopamine application, which also reduces horizontal cell coupling [43]–[45]. Here we show the hierarchy of horizontal cell length constant reduction for the three conditions: wild-type, wild-type with dopamine, and Cx57 knockout, calculated from ref. 28, Figs. 6a and 7b. For each condition, *red lines* indicate the median, *blue boxes* indicate the upper and lower quartiles; *black lines* indicate the data ranges, *black x*'s indicate two outliers. For comparison, mean horizontal cell dendritic tree diameter is 100 µm [28].
(0.16 MB TIF)

## References

[1] Enroth-Cugell C, Robson J (1966). The contrast sensitivity of retinal ganglion cells of the cat.. J Physiol.

[2] Shapley R, Lennie P (1985). Spatial frequency analysis in the visual system.. Annu Rev Neurosci.

[3] Rodieck R, Stone J (1965). Analysis of receptive fields of cat retinal ganglion cells.. J Neurophysiol.

[4] Barlow H (1953). Summation and inhibition in the frog's retina.. J Physiol.

[5] Kuffler S (1953). Discharge patterns and functional organization of mammalian retina.. J Neurophysiol.

[6] Sinclair J, Jacobs A, Nirenberg S (2004). Selective ablation of a class of amacrine cells alters spatial processing in the retina.. J Neurosci.

[7] Werblin F, Dowling J (1969). Organization of the retina of the mudpuppy, Necturus maculosus. II. Intracellular recording.. J Neurophysiol.

[8] Kaneko A (1970). Physiological and morphological identification of horizontal, bipolar and amacrine cells in goldfish retina.. J Physiol.

[9] Naka K, Witkovsky P (1972). Dogfish ganglion cell discharge resulting from extrinsic polarization of the horizontal cells.. J Physiol.

[10] Mangel S (1991). Analysis of the horizontal cell contribution to the receptive field surround of ganglion cells in the rabbit retina.. J Physiol.

[11] Yang X, Wu S (1991). Feedforward lateral inhibition in retinal bipolar cells: input-output relation of the horizontal cell-depolarizing bipolar cell synapse.. Proc Natl Acad Sci U S A.

[12] Fahey P, Burkhardt D (2003). Center-surround organization in bipolar cells: symmetry for opposing contrasts.. Vis Neurosci.

[13] Cook P, McReynolds J (1998). Lateral inhibition in the inner retina is important for spatial tuning of ganglion cells.. Nat Neurosci.

[14] Bieda M, Copenhagen D (1999). Sodium action potentials are not required for light-evoked release of GABA or glycine from retinal amacrine cells.. J Neurophysiol.

[15] Taylor W (1999). TTX attenuates surround inhibition in rabbit retinal ganglion cells.. Vis Neurosci.

[16] Flores-Herr N, Protti D, Wässle H (2001). Synaptic currents generating the inhibitory surround of ganglion cells in the mammalian retina.. J Neurosci.

[17] Roska B, Nemeth E, Orzo L, Werblin F (2000). Three levels of lateral inhibition: A space-time study of the retina of the tiger salamander.. J Neurosci.

[18] Barlow H, Fitzhugh R, Kuffler S (1957). Change of organization in the receptive fields of the cat's retina during dark adaptation.. J Physiol.

[19] Smirnakis S, Berry M, Warland D, Bialek W, Meister M (1997). Adaptation of retinal processing to image contrast and spatial scale.. Nature.

[20] Enroth-Cugell C, Shapley R (1973). Adaptation and dynamics of cat retinal ganglion cells.. J Physiol.

[21] Maffei L, Fiorentini A, Cervetto L (1971). Homeostasis in retinal receptive fields.. J Neurophysiol.

[22] Smith RA (1973). Luminance-dependent changes in mesopic visual contrast sensitivity.. J Physiol.

[23] Mangel S, Dowling J (1985). Responsiveness and receptive field size of carp horizontal cells are reduced by prolonged darkness and dopamine.. Science.

[24] Baldridge W, Ball A (1991). Background illumination reduces horizontal cell receptive-field size in both normal and 6-hydroxydopamine-lesioned goldfish retinas.. Vis Neurosci.

[25] Tornqvist K, Yang X, Dowling J (1988). Modulation of cone horizontal cell activity in the teleost fish retina. III. Effects of prolonged darkness and dopamine on electrical coupling between horizontal cells.. J Neurosci.

[26] Xin D, Bloomfield S (1999). Dark- and light-induced changes in coupling between horizontal cells in mammalian retina.. J Comp Neurol.

[27] Hombach S, Janssen-Bienhold U, Söhl G, Schubert T, Büssow H (2004). Functional expression of connexin57 in horizontal cells of the mouse retina.. Eur J Neurosci.

[28] Shelley J, Dedek K, Schubert T, Feigenspan A, Schultz K (2006). Horizontal cell receptive fields are reduced in connexin57-deficient mice.. Eur J Neurosci.

[29] Campbell F, Robson J (1968). Application of Fourier analysis to the visibility of gratings.. J Physiol.

[30] Stone C, Pinto L (1993). Response properties of ganglion cells in the isolated mouse retina.. Vis Neurosci.

[31] Bisti S, Clement R, Maffei L, Mecacci L (1977). Spatial frequency and orientation tuning curves of visual neurones in the cat: effects of mean luminance.. Exp Brain Res.

[32] Muller J, Dacheux R (1997). Alpha ganglion cells of the rabbit retina lose antagonistic surround responses under dark adaptation.. Vis Neurosci.

[33] Rodieck R (1965). Quantitative analysis of cat retinal ganglion cell response to visual stimuli.. Vision Res.

[34] Enroth-Cugell C, Robson J, Schweitzer-Tong D, Watson A (1983). Spatio-temporal interactions in cat retinal ganglion cells showing linear spatial summation.. J Physiol.

[35] Croner LJ, Kaplan E (1995). Receptive fields of P and M ganglion cells across the primate retina.. Vision Res.

[36] Prusky G, Alam N, Beekman S, Douglas R (2004). Rapid quantification of adult and developing mouse spatial vision using a virtual optomotor system.. Invest Ophthalmol Vis Sci.

[37] Douglas R, Alam N, Silver B, McGill T, Tschetter W (2005). Independent visual threshold measurements in the two eyes of freely moving rats and mice using a virtual-reality optokinetic system.. Vis Neurosci.

[38] Abdeljalil J, Hamid M, Abdel-Mouttalib O, Stéphane R, Raymond R (2005). The optomotor response: a robust first-line visual screening method for mice.. Vision Res.

[39] Benedek G, Benedek K, Kéri S, Letoha T, Janáky M (2003). Human scotopic spatiotemporal sensitivity: a comparison of psychophysical and electrophysiological data.. Doc Ophthalmol.

[40] Jensen R, Daw N (1984). Effects of dopamine antagonists on receptive fields of brisk cells and directionally selective cells in the rabbit retina.. J Neurosci.

[41] Jensen R, Daw N (1986). Effects of dopamine and its agonists and antagonists on the receptive field properties of ganglion cells in the rabbit retina.. Neuroscience.

[42] Vigh J, Witkovsky P (1999). Sub-millimolar cobalt selectively inhibits the receptive field surround of retinal neurons.. Vis Neurosci.

[43] Hampson E, Weiler R, Vaney D (1994). pH-gated dopaminergic modulation of horizontal cell gap junctions in mammalian retina.. Proc Biol Sci.

[44] He S, Weiler R, Vaney D (2000). Endogenous dopaminergic regulation of horizontal cell coupling in the mammalian retina.. J Comp Neurol.

[45] Teranishi T, Negishi K, Kato S (1983). Dopamine modulates S-potential amplitude and dye-coupling between external horizontal cells in carp retina.. Nature.

[46] Witkovsky P, Dearry A (1992). Functional roles of dopamine in the vertebrate retina.. Prog Ret Res.

[47] Wang Y, Mangel S (1996). A circadian clock regulates rod and cone input to fish retinal cone horizontal cells.. Proc Natl Acad Sci U S A.

[48] McMahon M, Packer O, Dacey D (2004). The classical receptive field surround of primate parasol ganglion cells is mediated primarily by a non-GABAergic pathway.. J Neurosci.

[49] Xia Y, Nawy S (2003). The gap junction blockers carbenoxolone and 18beta-glycyrrhetinic acid antagonize cone-driven light responses in the mouse retina.. Vis Neurosci.

[50] Packer O, Dacey D (2002). Receptive field structure of H1 horizontal cells in macaque monkey retina.. J Vis.

[51] Kamermans M, Fahrenfort I, Schultz K, Janssen-Bienhold U, Sjoerdsma T (2001). Hemichannel-mediated inhibition in the outer retina.. Science.

[52] Blakemore C, Campbell F (1969). On the existence of neurones in the human visual system selectively sensitive to the orientation and size of retinal images.. J Physiol.

[53] Cavonius C, Robbins D (1973). Relationships between luminance and visual acuity in the rhesus monkey.. J Physiol.

[54] Greenlee M, Georgeson M, Magnussen S, Harris J (1991). The time course of adaptation to spatial contrast.. Vision Res.

[55] DeValois RL, DeValois KK (1990). Spatial Vision..

[56] Atick JJ, Redlich AN (1992). What does the retina know about natural scenes?. Neural Comput.

[57] van Hateren J (1992). A theory of maximizing sensory information.. Biol Cybern.

[58] Vaney D (1991). Many diverse types of retinal neurons show tracer coupling when injected with biocytin or Neurobiotin.. Neurosci Lett.

[59] Urschel S, Höher T, Schubert T, Alev C, Söhl G (2006). Protein kinase A-mediated phosphorylation of connexin36 in mouse retina results in decreased gap junctional communication between AII amacrine cells.. J Biol Chem.

[60] Smith R (1995). Simulation of an anatomically defined local circuit: the cone-horizontal cell network in cat retina.. Vis Neurosci.

[61] Nirenberg S, Carcieri S, Jacobs A, Latham P (2001). Retinal ganglion cells act largely as independent encoders.. Nature.

[62] Lyubarsky AL, Daniele LL, Pugh EN (2004). From candelas to photoisomerizations in the mouse eye by rhodopsin bleaching in situ and the light-rearing dependence of the major components of the mouse ERG.. Vision Res.

[63] Lyubarsky AL, Falsini B, Pennesi ME, Valentini P, Pugh ENJ (1999). UV- and midwave-sensitive cone-driven retinal responses of the mouse: a possible phenotype for coexpression of cone photopigments.. J Neurosci.

[64] Shelley J Personal communication.

[65] Dodd RL (1998). The role of arrestin and recoverin in signal transduction by retinal rod photoreceptors..

[66] Wichmann F, Hill N (2001). The psychometric function: I. Fitting, sampling, and goodness of fit.. Percept Psychophys.

[67] Lucas RJ (2003). Diminished pupillary reflex at high irradiances in melanopsin-knockout mice.. Science.

[68] Saszik SM, Robson JG, Frishman LJ (2002). The scoptopic threshold response of the dark-adapted electroretinogram of the mouse.. J. Physiol..

[69] Umino Y, Solessio E, Barlow RB (2008). Speed, spatial, and temporal tuning of rod and cone vision in mouse.. J. Neurosci..

