# Supplementary figures and images for: Ganglion Cell Adaptability: Does the Coupling of Horizontal Cells Play a Role?
Source: PLoS One. 2008 Mar 5;3(3):e1714. doi: 10.1371/journal.pone.0001714 (PMC2246161; doi:10.1371/journal.pone.0001714)

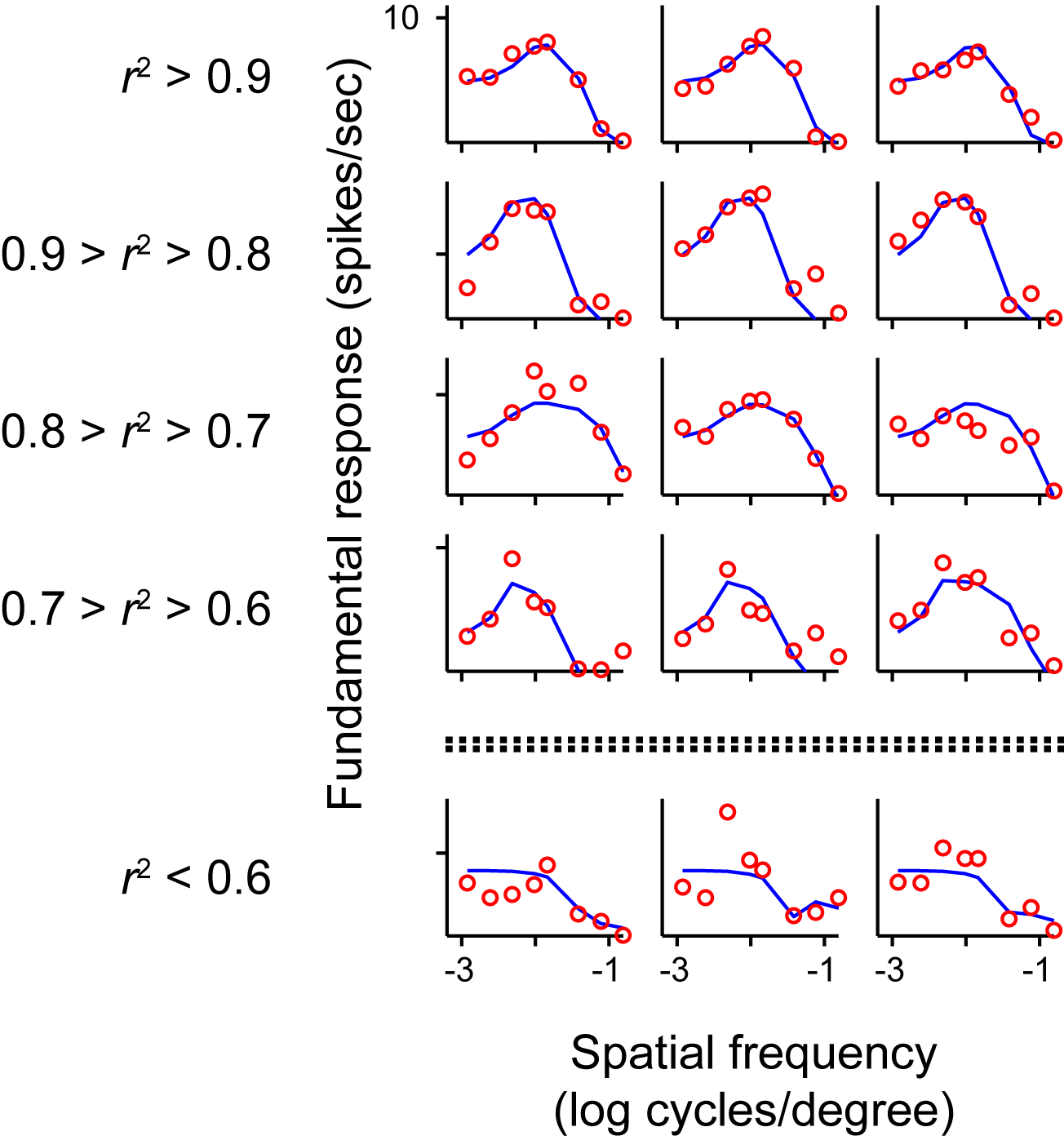

Supplement: Figure S1 — As indicated in the main text ( Methods ), for quality control, and for consistency with previous work [6], only fits with r2 values >0.6 were used. To provide intuition for the quality of an r2 value of >0.6, a series of fits from r2>0.9 to r2<0.6 is shown. A natural breakdown begins below 0.6. Data are plotted on semi-log plots; red dots indicate cells' responses, blue curves indicate fits. (0.31 MB TIF) [file pone.0001714.s001.tif]

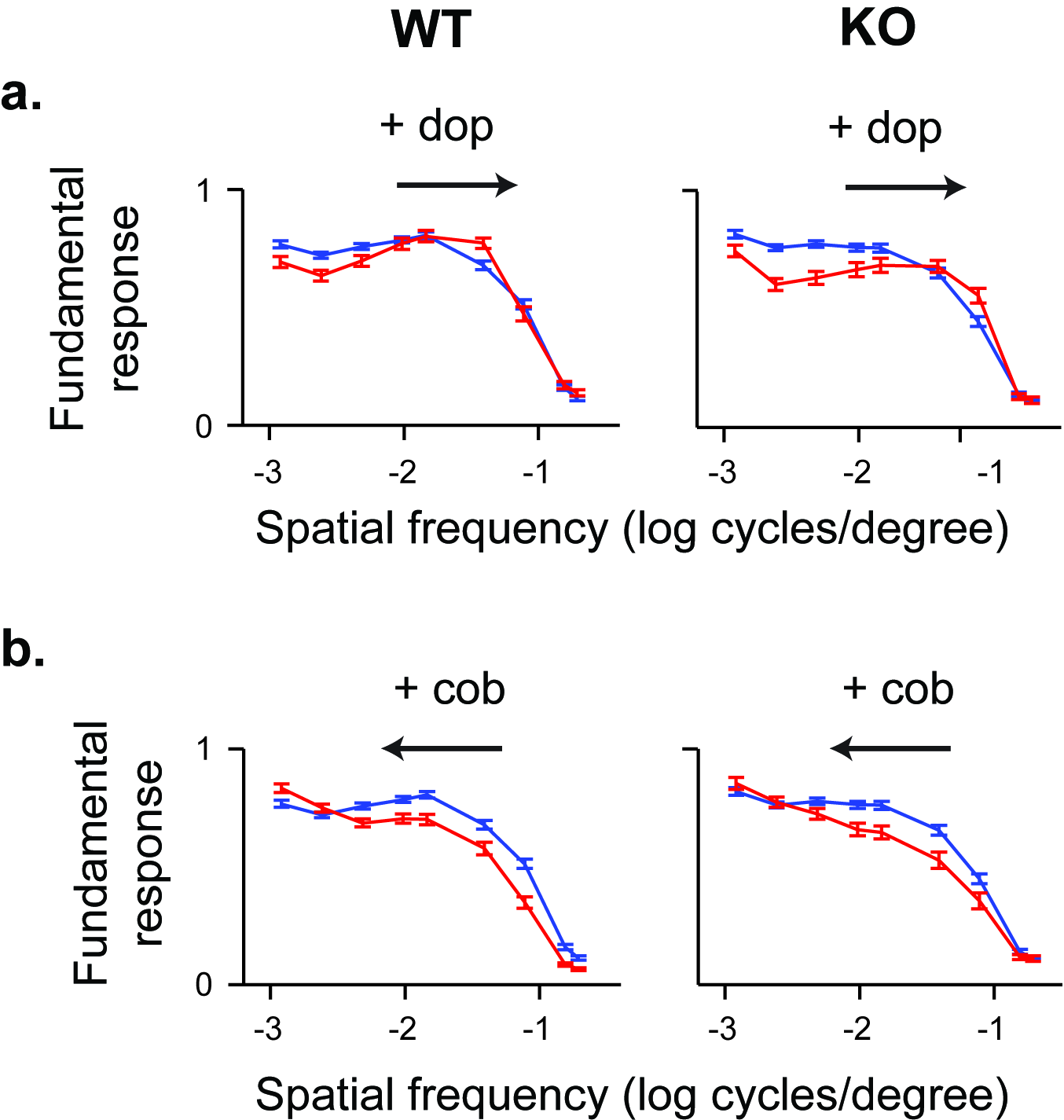

Supplement: Figure S2 — Shifts in spatial tuning following dopamine and cobalt application, presented as average tuning curves. In the main text, the shifts were presented as center-of-mass distributions; that is, we took each cell's tuning curve, measured its center of mass and presented the distribution of center of mass values for all cells in the data set (see Figs. 5 and 6). For the interested reader, we show here the shifts as average tuning curves (mean±SEM); arrows indicate direction of shift. Consistent with the center of mass analysis, where all significance tests are presented, dopamine causes a shift to the right for both genotypes, and cobalt causes a shift to the left for both genotypes. Blue indicates no drug; red indicates drug. (0.38 MB TIF) [file pone.0001714.s002.tif]

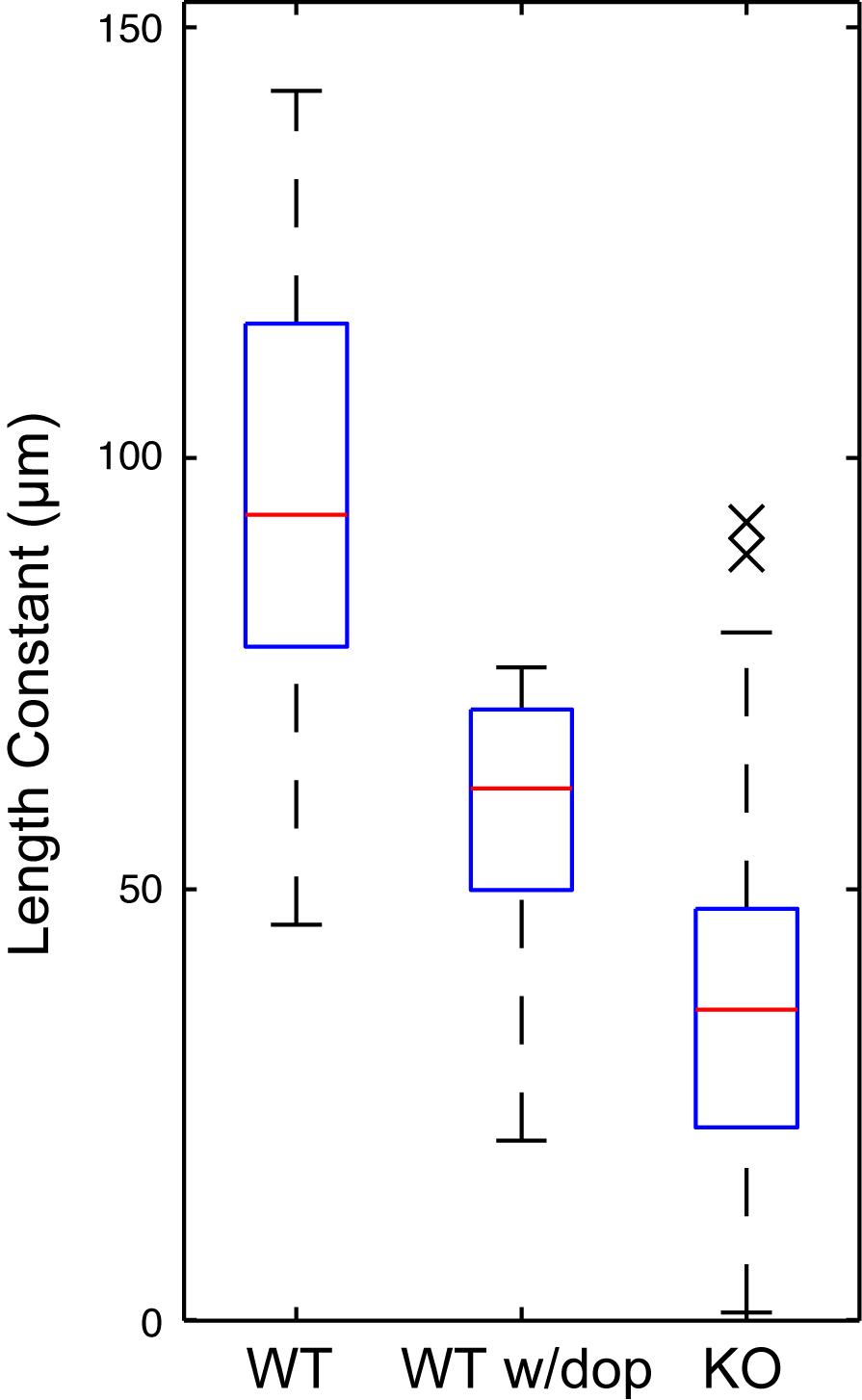

Supplement: Figure S3 — Horizontal cell length constants in the Cx57-deficient mice are significantly reduced. As indicated in the main text, the evidence that knocking out Cx57 blocks horizontal cell coupling is that dye spread (neurobiotin) is >99% abolished, and horizontal cell length constants are significantly reduced, with the reduction greater than that produced by dopamine application, which also reduces horizontal cell coupling [43]–[45]. Here we show the hierarchy of horizontal cell length constant reduction for the three conditions: wild-type, wild-type with dopamine, and Cx57 knockout, calculated from ref. 28, Figs. 6a and 7b. For each condition, red lines indicate the median, blue boxes indicate the upper and lower quartiles; black lines indicate the data ranges, black x's indicate two outliers. For comparison, mean horizontal cell dendritic tree diameter is 100 µm [28]. (0.16 MB TIF) [file pone.0001714.s003.tif]
